# Factors That Affect the Rates of Adaptive and Nonadaptive Evolution at the Gene Level in Humans and Chimpanzees

**DOI:** 10.1093/gbe/evac028

**Published:** 2022-02-15

**Authors:** Vivak Soni, Adam Eyre-Walker

**Affiliations:** School of Life Sciences, University of Sussex, Brighton, United Kingdom

**Keywords:** adaptive evolution, humans, chimpanzees, recombination rate, gene age

## Abstract

The rate of amino acid substitution has been shown to be correlated to a number of factors including the rate of recombination, the age of the gene, the length of the protein, mean expression level, and gene function. However, the extent to which these correlations are due to adaptive and nonadaptive evolution has not been studied in detail, at least not in hominids. We find that the rate of adaptive evolution is significantly positively correlated to the rate of recombination, protein length and gene expression level, and negatively correlated to gene age. These correlations remain significant when each factor is controlled for in turn, except when controlling for expression in an analysis of protein length; and they also generally remain significant when biased gene conversion is taken into account. However, the positive correlations could be an artifact of population size contraction. We also find that the rate of nonadaptive evolution is negatively correlated to each factor, and all these correlations survive controlling for each other and biased gene conversion. Finally, we examine the effect of gene function on rates of adaptive and nonadaptive evolution; we confirm that virus-interacting proteins (VIPs) have higher rates of adaptive and lower rates of nonadaptive evolution, but we also demonstrate that there is significant variation in the rate of adaptive and nonadaptive evolution between GO categories when removing VIPs. We estimate that the VIP/non-VIP axis explains about 5–8 fold more of the variance in evolutionary rate than GO categories.

Significance

The rate at which a protein evolves depends on a number of factors including its age, length, and expression level, as well as its function and recombination rate. However, these patterns might be due to either adaptive or nonadaptive evolution. We analyze the rate at which proteins evolve between humans and chimpanzees and show that rates of both adaptive and nonadaptive evolution are affected by multiple factors, suggesting that the rate at which a protein evolves is due to a complex set of interacting variables.

## Introduction

There is substantial variation in the rate of evolution between different genes within a genome; some genes, such as those coding for histones, evolve very slowly, whereas many genes involved in immunity evolve rapidly (Clark et al. 2003; Chimpanzee Sequencing and Analysis Consortium 2005; Nielsen et al. 2005; Sackton et al. 2007; Obbard et al. 2009). The reasons for this variation have been extensively studied and a number of factors appear to influence or be correlated to the rate of protein evolution including function (Pröschel et al. 2006; Haerty et al. 2007; Obbard et al. 2009), mutation rate (Taddei et al. 1997; Tenaillon et al. 1999; Giraud et al. 2001; Denamur and Matic 2006; Lynch et al. 2016), recombination rate (RR) (Hill and Robertson 1966; Marais and Charlesworth 2003), gene expression (Pál et al. 2001; Subramanian and Kumar 2004; Wright et al. 2004; Lemos et al. 2005), and protein length (Zhang 2000; Lipman et al. 2002; Liao et al. 2006). Correlations with other factors, such as essentiality, appear to be less clear (Hurst and Smith 1999). Any one of these patterns could be due to adaptive or nonadaptive evolution, but the relative roles of these two different evolutionary processes have rarely been studied. Note, that we define advantageous mutations as those that on average increase in frequency and are subject to either natural and sexual selection.

At the functional level, genes involved in immunity, tumor suppression, apoptosis, and spermatogenesis have been shown to have higher rates of adaptive evolution in hominids (Clark et al. 2003; Chimpanzee Sequencing and Analysis Consortium 2005; Nielsen et al. 2005). Particularly striking is the amount of adaptive evolution that appears to occur in virus-interacting genes, which appear to account for 30% of all adaptive substitutions in hominids, whereas these genes only constitute 13% of the proteome by length (Enard et al. 2016). In *Drosophila*, it has been shown that male-biased genes, such as testes specific genes, have higher rates of adaptive evolution (Pröschel et al. 2006; Haerty et al. 2007), as do genes involved in immunity (Sackton et al. 2007; Obbard et al. 2009). The dominant role of viral interacting proteins (VIPs) in hominid adaptive evolution begs the question of whether there is variation between other categories of genes, and how much of the variation in the rate of adaptive evolution is partitioned between the VIP and non-VIP categories. The role of gene function in determining nonadaptive evolution has not been addressed in detail.

The rate of protein sequence evolution has been shown to be correlated to gene expression, with highly expressed genes having lower rates of protein evolution in both eukaryotes (Pál et al. 2001; Subramanian and Kumar 2004; Wright et al. 2004; Lemos et al. 2005) and prokaryotes (Rocha and Danchin 2004). Moutinho et al. (2019) has shown that this correlation is due to both adaptive and nonadaptive evolution in *Drosophila* suggesting that gene expression constrains the rate of adaptive substitution as well as the effect of purifying selection. In *Arabidopsis* the correlation with expression seems to be largely associated with nonadaptive evolution (Moutinho et al. 2019). The role of gene length has also been studied, with several studies showing that smaller genes evolve more rapidly (Zhang 2000; Lipman et al. 2002; Liao et al. 2006). Again, this appears to be due to both adaptive and nonadaptive evolution, in *Drosophila* species, but possibly only due to nonadaptive evolution in *Arabidopsis* (Moutinho et al. 2019).

Genes differ not only in function, expression, and length, but also in age (Lynch 2002; Daubin and Ochman 2004; Tautz and Domazet-Lošo 2011; Neme and Tautz 2013). Multiple studies have shown that young genes (i.e., those genes whose recognized homologs are only present in closely related species; Domazet-Loso et al. 2007) evolve faster than old genes (Thornton and Long 2002; Domazet-Loso and Tautz 2003; Krylov et al. 2003; Daubin and Ochman 2004; Albà and Castresena 2005; Wang et al. 2005; Cai et al. 2006; Wolf et al. 2009; Cai and Petrov 2010; Vishnoi et al. 2010; Zhang et al. 2010; Tautz and Domazet-Lošo 2011; Cui et al. 2015). Cai and Petrov (2010) found clear evidence for the role of nonadaptive evolution in this relationship but no evidence for adaptive evolution. However, there is an expectation that young genes will be further from their evolutionary optimum than old genes, and hence that they should undergo rapid adaptive evolution when they are born. There is some limited evidence for this; the *jingwei* gene, which appeared very recently in the *Drosophila* phylogeny is evolving very rapidly, with 80% of the amino acid substitutions estimated to have been due to adaptive evolution (Long and Langley 1993).

Recombination is expected to affect the probability that both advantageous and deleterious mutations are fixed, due to its ability to reduce Hill–Robertson interference between selected mutations (Hill and Robertson 1966; Marais and Charlesworth 2003). Rates of adaptation have been shown to be strongly positively correlated to RR in *Drosophila* (Presgraves 2005; Betancourt et al. 2009; Arguello et al. 2010; Mackay et al. 2012; Campos et al. 2014; Castellano et al. 2016; Moutinho et al. 2019) and *Arabidopsis* (Moutinho et al. 2019), and rates of nonadaptive evolution to be negatively correlated in both *Drosophila* and *Arabidopsis* species (Moutinho et al. 2019).

In summary, a number of factors have been shown to correlate to rates of protein evolution, and in some of these cases the relative roles of adaptive and nonadaptive evolution have been disentangled. However, relatively little work has been done on these questions in hominids. We addressed these questions by considering the role of gene age, RR, gene expression, protein length, and gene function in determining rates of both adaptive and nonadaptive evolution. To disentangle the effects of adaptive and nonadaptive evolution, we use an extension of the McDonald–Kreitman test which estimates these quantities taking into account the distribution fitness effects of new mutations.

## Results

We set out to investigate whether a number of gene-level factors affect the rate of adaptive and nonadaptive evolution in hominids—the RR, gene age, the level of gene expression, gene length, and gene function. We measure the rates of adaptive and nonadaptive evolution using the statistics ω_a_ and ω_na_, which are estimates of the rate of evolution relative to the mutation rate. We estimated both statistics using an extension of the McDonald–Kreitman method, in which the pattern of substitution and polymorphism at neutral and selected sites is used to infer the rates of substitution, taking into account the influence of slightly deleterious mutations. We use the method implemented in Grapes (Galtier 2016), which is a maximum likelihood implementation of the second method proposed by Eyre-Walker and Keightley (2009). Estimating rates of adaptive and nonadaptive evolution in individual genes is impractical, as most genes have relatively little polymorphism data. We therefore group genes together, according to the factors analyzed.

We estimated ω_a_ and ω_na_ using 16,344 genes for the divergence between humans and chimpanzees using African SNPs from the 1000 genomes data (1000 Genomes Project Consortium 2015). We find that the average rate of adaptive evolution is approximately 5-fold lower than the rate of nonadaptive evolution (ω_a_=0.037 [95% CIs estimates using bootstrapping 0.035 and 0.039] vs. ω_na_=0.19 [0.19,0.19]). The proportion of substitutions that are adaptive, α, is estimated to be 0.16, which is close to previous recent estimates (Boyko et al. 2008; Eyre-Walker and Keightley 2009; Messer and Petrov 2013).

### Adaptive Evolution

**Fig. 1.**
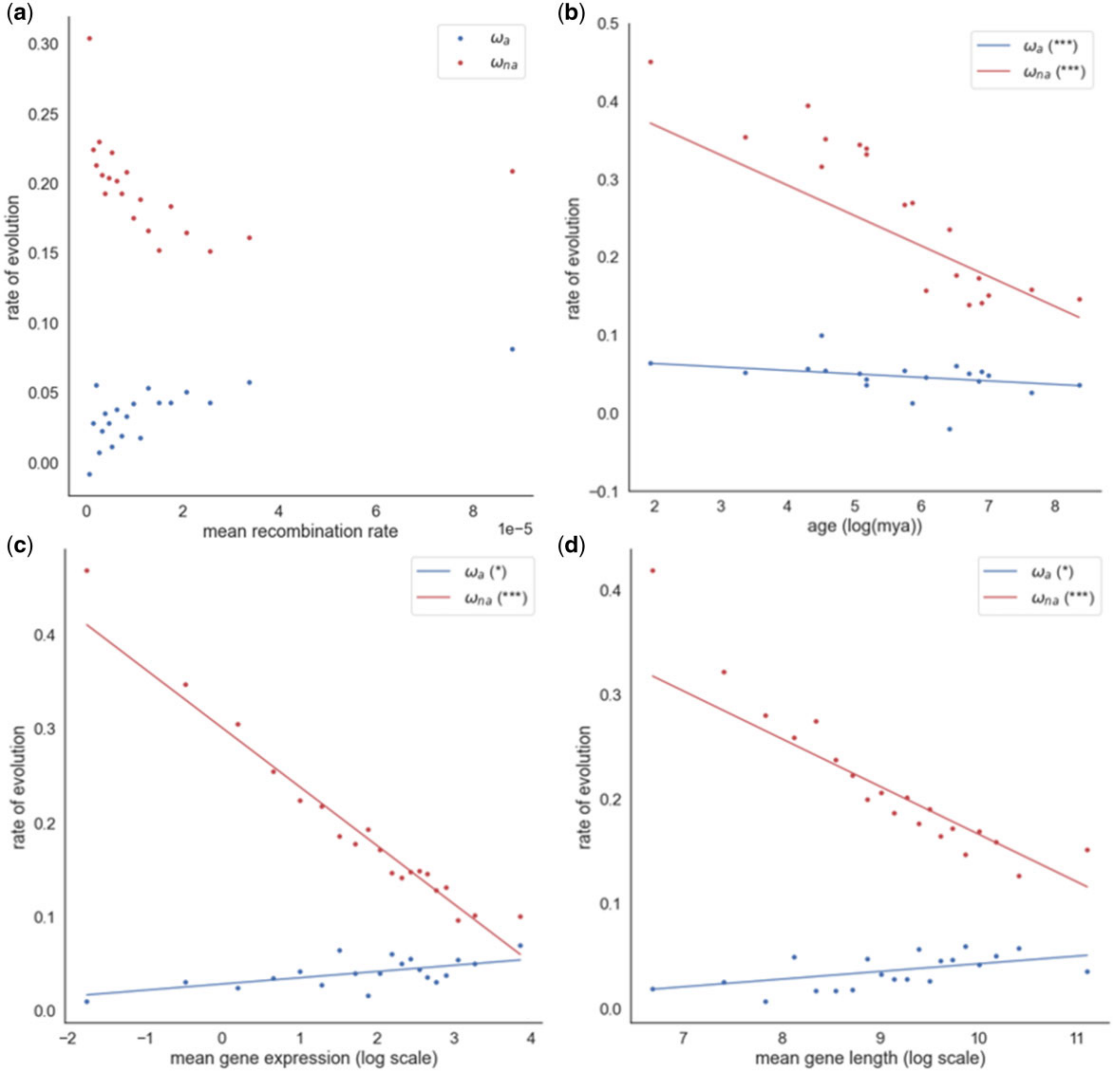
Estimates of ω_a_ and ω_na_ plotted against the (*a*) mean RR, *(b)* gene age, (*c*) mean gene expression, and (*d*) mean protein length. The respective significance of each correlation is shown in the plot legend, (*P < 0.05; **P < 0.01; ***P < 0.001; “.” 0.05 ≤ *P* < 0.10 for ω_a_ and ω_na_). Also shown is the line of best fit through the data. An unweighted regression is fitted to the estimates of ω_a_ and ω_na_.

The rate of adaptation is expected to be retarded in regions of low recombination because of Hill–Robertson interference, and we do indeed find that the rate of adaptive evolution is significantly positively correlated to the rate of recombination in hominids (fig. 1*a*;*r* = 0.74, *P* < 0.001); this correlation is also significant if we use pedigree, rather than population genetic estimates of the RR (*r*=−0.48, *P* = 0.033). A similar positive correlation has previously been observed in *Drosophila* (Presgraves 2005; Betancourt et al. 2009; Arguello et al. 2010; Mackay et al. 2012; Campos et al. 2014; Castellano et al. 2016). In the most detailed study of this relationship in *Drosophila*, Castellano et al. (2016) found that the rate of adaptive evolution increases with RR, but that it asymptotes, suggesting that above a certain level of recombination, Hill–Robertson interference has little effect. It is not clear whether there is an asymptote in humans (fig. 1*a*); the rate of increase in the rate of adaptive evolution with RR does appear to decrease, but not sufficiently to declare that there is an asymptote. The same pattern is evident if we divide the data up into 50 instead of 20 bins (*r* = 0.58, *P* < 0.001) (supplementary fig. S1, Supplementary Material online). Unfortunately, we have relatively few genes with high RRs.

Young genes have been shown to evolve faster than old genes (Thornton and Long 2002; Domazet-Loso and Tautz 2003; Krylov et al. 2003; Daubin and Ochman 2004; Albà and Castresena 2005; Wang et al. 2005; Cai et al. 2006; Wolf et al. 2009; Cai and Petrov 2010; Vishnoi et al. 2010; Zhang et al. 2010; Tautz and Domazet-Lošo 2011; Cui et al. 2015). There is an expectation that young genes will undergo faster rates of adaptive evolution because they are further from their adaptive optima (Wright 1931, 1932), and we do indeed find a significant negative correlation between ω_a_ and gene age in hominids (*r*=−0.40, *P* = 0.012) (fig. 1*b*).

Highly expressed genes have been shown to exhibit lower rates of protein evolution in both eukaryotes (Pál et al. 2001; Subramanian and Kumar 2004; Wright et al. 2004; Lemos et al. 2005) and prokaryotes (Rocha and Danchin 2004). Moutinho et al. (2019) found significant negative correlations in *Drosophila* species between ω_a_ and both gene expression and protein length. Intriguingly, the correlations are reversed in hominids, with both correlations being significantly positive (gene expression: *r* = 0.642, *P* = 0.002; protein length: *r* = 0.597, *P* = 0.005) (fig. 1*c* and *d*).

### Independent Effects

Our measure of adaptive evolution, ω_a_, is significantly positively correlated to RR, expression, and protein length, and negatively to gene age. However, the rate of recombination, gene age, gene expression, and protein length are all significantly, or nearly significantly, correlated to each other (table 1) so it is important to determine whether each factor has an independent effect on the rate of adaptive evolution; that is, the correlation between Y and X, might be due to the fact that each is correlated to a third factor Z, and with no variation in Z there is no correlation between Y and X. To investigate this, we conducted two analyses. In the first instance, we repeated our analyses controlling for each factor in turn by taking the values of the co-correlate around the modal value—we took the modal value and 0.5 standard deviations (SDs) either side. This significantly reduced the coefficient of variation (CV) of the co-correlate within each analysis, largely controlling for this factor (table 1). However, controlling for each factor this way reduces the data set considerably, so we also ran an analysis in which we calculated the expected correlation between two variables under the assumption that they are correlated solely because of their correlation to a third variable. It can be shown (see Materials and Methods) that if the correlation between Y and Z is *r*_YZ_ and that between X and Z is *r*_XZ_, then expected correlation between Y and X due to the covariation with Z is $r_{\mathrm{YX}}=\mathrm{Sign}\;\sqrt{r_{\mathrm{YZ}}^{2}\;r_{\mathrm{XZ}}^{2}}$, where Sign is positive if both *r*_YZ_ and *r*_XZ_ are positive or negative, and negative otherwise. In both analyses, we only investigate factors that could generate an artifactual correlation of the correct sign.

**Table 1:** The Correlation between Gene Age, Gene Expression, Protein Length, and RR

	Gene Expression	Protein Length	RR	CV	CV of Near Modal Values
Gene age	0.87***	0.86***	−0.62**	1.4	0.38
Gene expression		0.44***	−0.035***	1.5	0.41
Protein length			0.10***	1.7	0.50
RR				1.1	0.33

Note.—Logs were taken of all variables. The CV column is the coefficient of variation of the factor for all the data. The final column is the CV of the restricted data (i.e., when we control for the factor in question by restricting the analysis to genes with the modal value ±0.5 SDs).

* *P* < 0.05, **P < 0.01, ***P < 0.001.

Our two analyses suggest that there is a direct association between ω_a_ and RR; when we control for age and length, we find that although the correlation is no longer significant when we control for either variable, the correlation does remain positive, and the observed correlations are significantly greater than the predicted correlation (table 2). The analysis also suggests that there is a direct association between ω_a_ and age, because the correlation remains significantly negative when we control for RR, and the predicted correlation is significantly smaller in magnitude than the observed correlation. However, the results with gene expression and length are less clear; when each variable is controlled for in the analysis of the other, the correlation becomes nonsignificant (table 2). The observed correlation between ω_a_ and expression is significantly greater than the predicted correlation, using length as the covariate, whereas the opposite is not true; this would seem to suggest that there is a direct correlation between ω_a_ and expression, and that the correlation between ω_a_ and length may be due to the fact that both are correlated to expression. However, the evidence is not strong in support of this hypothesis.

**Table 2:** The Observed Correlation between Y and X Controlling for a Covariate, Z, and the Observed and Predicted Correlation between Y and X Assuming the Relationship Is Solely due to the Correlation between Each Variable and a Third Factor Z

Y Variate	X Variate	Observed *r*	Z Variate	Observed *r*—Controlling for Z	Predicted *r*	Predicted/Observed>1
ω_a_	RR	0.74***	Age	0.25	0.15	0
ω_a_	RR	0.74***	Length	0.43	0.086	0
ω_a_	Age	−0.40*	RR	−0.58*	−0.093	0.02
ω_a_	Expression	0.64**	Length	0.00	0.38	0.03
ω_a_	Length	0.60**	RR	0.64**	0.091	0
ω_a_	Length	0.60**	Expression	0.25	0.37	0.13
ω_na_	RR	−0.73***	Length	−0.54*	−0.34	0
ω_na_	Age	−0.91***	Expression	−0.76**	−0.76	0
ω_na_	Age	−0.91***	Length	−0.87***	−0.75	0
ω_na_	Expression	−0.98***	Age	−0.74***	−0.90	0
ω_na_	Expression	−0.98***	Length	−0.61**	−0.95	0.01
ω_na_	Length	−0.94***	RR	−0.91***	−0.42	0
ω_na_	Length	−0.94***	Age	−0.49*	−0.88	0
ω_na_	Length	−0.94***	Expression	−0.71***	−0.89	0

Note.—The final column gives the proportion of 100 bootstrap replicates in which the predicted correlation divided by the observed correlation is greater than 1—that is, the predicted correlation is larger in magnitude.

* *P* < 0.05, **P < 0.01, ***P < 0.001.

### Controlling for Rate in Age Analysis

There is another factor that needs to be controlled for in any analysis of age—fast evolving genes are harder to identify in more distant species (Weisman et al. 2020), and this can lead to an artifactual correlation between the age of a gene and the rate of evolution because gene age is underestimated in fast evolving genes. To try and control for this effect, we reduced our data set to those genes around the modal value of d*N*. The distribution of nonsynonymous substitution rates is bimodal, with many genes having d*N*=0. We took genes around the second mode, those with rates between 0.002 and 0.007. This reduces our data set from 15,439 to 4,961 genes, and as a consequence, we had to combine multiple age categories together. We find no significant correlation between ω_a_ and age when we do this (*r* = 0.41, *P* = 0.27), suggesting that the correlation between ω_a_ and age might be an artifact of the problems in identifying fast evolving genes in older taxa.

### Controlling for Biased Gene Conversion

Biased gene conversion (BGC) can potentially impact estimates of the rate of adaptive evolution, either by increasing the fixation probability of S over W neutral alleles (Galtier and Duret 2007; Berglund et al. 2009; Ratnakumar et al. 2010; Rousselle et al. 2020), or by promoting the fixation of slightly deleterious S alleles (Duret and Galtier 2009; Glémin 2010; Necşulea et al. 2011; Lachance and Tishkoff 2014; Rousselle et al. 2020). To investigate whether BGC affects our results, we can leverage some of the results above. The correlation between ω_a_ and either age and protein length remains significant if we control for RR (table 2) (supplementary figs. S3*a* and S6*a*, Supplementary Material online, respectively), suggesting that BGC is unlikely to be responsible for these correlations. If we control for RR in the regression between ω_a_ and expression, we find that the correlation remains, suggesting that this correlation is also not due to BGC (*r* = 0.78, *P* < 0.001) (supplementary fig. S5*a*, Supplementary Material online).

To investigate whether the correlation between ω_a_ and RR is due to BGC, we performed a different analysis restricting the analysis to those polymorphisms and substitutions that are unaffected by BGC—that is, A<>T and G<>C changes. This reduces our data set to about 20% of its previous size. We find that there is still a positive correlation, although it is no longer significant (*r* = 0.10, *P* = 0.093) (supplementary fig. S2, Supplementary Material online).

In conclusion, ω_a_ is positively correlated to RR, protein length, and gene expression level, and to a large extent these correlations survive controlling for each other and BGC; the exceptions are protein length when expression is controlled for, and the positive relationship between ω_a_ and RR when BGC is controlled for.

### Nonadaptive Evolution

We repeated the analysis above for the rate of nonadaptive evolution. We find that ω_na_ is highly significantly negatively correlated to RR (whether we use population genetic or pedigree estimates), gene age, length, and expression (fig. 1). All of these correlations remain significant when controlling for potentially confounding factors, and the observed correlation is significantly greater in magnitude than the predicted correlation (table 2). Hence, we can conclude that all four factors have significant independent effects on ω_na_. As with the analysis of ω_a_ it is possible that these correlations are due to BGC. However, if we control for RR in our analyses, we find that all the negative correlations persist (gene age: *r*=−0.89, *P* < 0.001; gene length: *r*=−0.91, *P* < 0.001; gene expression: *r* = 0.99, *P* < 0.001). In the case of the correlation between ω_na_ and RR, if we restrict the analysis to G<>C and A<>T mutations we find that ω_na_ remains significantly negatively correlated to RR (*r*=−0.65, *P* < 0.001). If we control for the rate of evolution in the analysis of age by using genes with d*N* values around the modal value, as we did for ω_a_, we find the correlation between ω_na_ and gene age remains significant (*r* = −0.72, *P* = 0.027).

### Gene Function

**Fig. 2.**
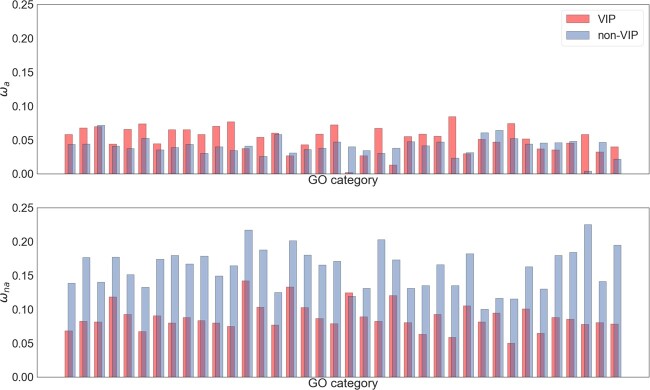
Estimates of ω_a_ (top) and ω_na_ (bottom) for GO categories that contain >100 VIP and non-VIP genes.

In the second part of our analysis, we consider the effect of gene function on the rate of adaptive and nonadaptive evolution. It has previously been demonstrated that genes whose products interact with viruses—VIPs—have higher rates of adaptive evolution than other genes in primates (Enard et al. 2016). We confirm this pattern. In our analysis, in which we have used a different method and statistic to estimate the rate of adaptive evolution, we find that the rate of adaptive evolution among VIPs is approximately 40% greater than in non-VIPs (ω_a_=0.052 vs. 0.032), a difference that is highly significant (*P* < 0.001). This pattern is consistent across almost all GO categories that have at least 100 genes, supporting the results of Enard et al. (2016) (fig. 2).

It is evident however, that there is substantial variation between GO categories for non-VIP genes, and this variation is significant, taking into account that individual genes can contribute to multiple GO categories (*P* = 0.0012). This pattern is replicated if we include GO categories which do not include VIP proteins (*P* = 0.0010). The GO categories which have the highest rate of adaptive evolution are ubiquitin protein ligase binding, and protein kinase binding (table 3).

**Table 3:** Top Ten GO Categories, Ranked by Rate of Adaptive Substitution

GO Category	ω_a_	ω_a_ 95% CIs
Ubiquitin protein ligase binding	0.0843	0.0702–0.0995
Protein kinase binding	0.0804	0.0698–0.0914
Sequence-specific DNA binding	0.0735	0.0633–0.0842
DNA-binding transcription factor activity	0.0719	0.0628–0.0812
Transcription factor complex	0.0682	0.0496–0.0883
Transcription by RNA polymerase II	0.0673	0.0518–0.0836
Negative regulation of apoptotic process	0.0671	0.0552–0.0796
Chromatin organization	0.0669	0.0567–0.0775
DNA-binding transcription activator activity	0.0649	0.0524–0.078
Transcription coactivator activity	0.0648	0.0519–0.0786

What are the relative contributions of GO category and VIP status to the variation in the rate of adaptive evolution—that is, is most of the variation in the rate of adaptive evolution due to whether the gene encodes a VIP or not, or is most of the variation due to other functional considerations? To investigate this, we performed a two-way analysis of variance on ω_a_ and estimated the variance components. We find that the distinction between VIP and non-VIP contributes approximately 5× the variance in ω_a_ as the variation between GO categories, suggesting that whether a gene encodes a VIP has a major effect on its rate of adaptation (supplementary table S1, Supplementary Material online).

But what of nonadaptive evolution? If we divide our data into genes that interact with viruses and those that do not, we find that rates of nonadaptive evolution are substantially higher in non-VIP genes (ω_na_=0.198 vs. 0.101). As Enard et al. (2016) found, this pattern is replicated across GO categories (fig. 2). There is substantial and significant variation in ω_na_ across GO categories excluding VIP genes, taking into account that individual genes can contribute to multiple GO categories (*P* < 0.001). This pattern is replicated if we include GO categories which do not include VIP proteins (*P* < 0.001). The GO categories that have the highest non-VIP rates of nonadaptive evolution are both related to immune system response (table 4). If we partition the variance between VIP/non-VIP and GO categories, we find that the distinction between VIP and non-VIP contributes over 8× the variance in ω_na_ as the variation between GO categories, suggesting that whether a gene encodes a VIP has a major effect on its rate of nonadaptive evolution (supplementary table S2, Supplementary Material online) as well as its rate of adaptation.

**Table 4:** Top Ten GO Categories, Ranked by Rate of Nonadaptive Substitution

GO Category	ω_na_	ω_na_ 95% CIs
Immune system process	0.297	0.283–0.310
Innate immune response	0.264	0.248–0.279
Chromosome	0.262	0.249–0.274
Protein C-terminus binding	0.246	0.228–0.264
Centrosome	0.243	0.232–0.253
DNA repair	0.236	0.223–0.249
Signal transduction	0.225	0.219–0.231
Neutrophil degranulation	0.218	0.206–0.229
Extracellular region	0.217	0.211–0.223
Proteolysis	0.204	0.195–0.214

## Discussion

It has been previously shown that the rate of evolution correlates to a number of factors including RR (Presgraves 2005; Betancourt et al. 2009; Arguello et al. 2010; Mackay et al. 2012; Campos et al. 2014; Castellano et al. 2016; Moutinho et al. 2019), gene age (Thornton and Long 2002; Domazet-Loso and Tautz 2003; Krylov et al. 2003; Daubin and Ochman 2004; Albà and Castresena 2005; Wang et al. 2005; Cai et al. 2006; Wolf et al. 2009; Cai and Petrov 2010; Vishnoi et al. 2010; Zhang et al. 2010; Tautz and Domazet-Lošo 2011; Cui et al. 2015), expression level (Pál et al. 2001; Rocha and Danchin 2004; Subramanian and Kumar 2004; Wright et al. 2004; Lemos et al. 2005; Moutinho et al. 2019), and protein length (Zhang 2000; Lipman et al. 2002; Liao et al. 2006; Moutinho et al. 2019). In addition, the rate of evolution has been shown to vary with gene function (Clark et al. 2003; Chimpanzee Sequencing and Analysis Consortium 2005; Nielsen et al. 2005). In this study, we have correlated each of these factors to ω_a_ and ω_na_ in hominids, allowing us to disentangle the effects of adaptive and nonadaptive evolution. We find that ω_a_ is correlated to all four factors, and that when we control for each factor in turn, there is evidence for an independent influence of RR, gene age, and gene expression. These correlations generally remain when controlling for the effects of BGC, although the relationship with RR is not significant. However, the correlation with gene age could be an artifact of fast evolving genes having higher rates of adaptive evolution and being more difficult to identify in older taxa; when we control for the rate at which a protein evolves, the negative correlation between ω_a_ and gene age becomes nonsignificant consistent with this possibility.

In contrast, we find that all four factors have significant independent effects on ω_na_, and that all of these remain significant when we control for each in turn, and control for BGC. Several studies on both eukaryotes (Pál et al. 2001; Subramanian and Kumar 2004; Wright et al. 2004; Lemos et al. 2005; Moutinho et al. 2019) and prokaryotes (Rocha and Danchin 2004) have demonstrated that more highly expressed genes have lower rates of protein sequence evolution. Our results support these previous findings, with the negative correlation between ω_na_ and gene expression suggesting that more highly expressed genes are under greater constraint in hominids. Drummond et al. (2005) suggest a general hypothesis that more highly expressed genes evolve slowly (i.e., are under higher selective constraint) because of the selection against the expression level cost of protein misfolding, wherein selection acts by favoring protein sequences that accumulate less translational missense errors. We also find a significant negative correlation between ω_na_ and gene length. This supports former studies that have shown that smaller genes evolve more rapidly (Zhang 2000; Lipman et al. 2002; Liao et al. 2006; Moutinho et al. 2019), suggesting that smaller protein-coding regions are under more relaxed purifying selection.

### Methodological Concerns

The method we have used to infer ω_a_ and ω_na_ makes a number of simplifying assumptions. We assume that the DFE is well described by a gamma distribution, which does appear to fit the SFS spectra well in analyses comparing different functional forms of the DFE in hominids (Boyko et al. 2008; Galtier and Rousselle 2020). We have also assumed that new nonsynonymous mutations are either deleterious or strongly advantageous. However, there are likely to be slightly advantageous mutations and these can lead to an overestimate of the rate of adaptive evolution (Tataru et al. 2017). It is therefore possible that the correlations we have observed are not necessarily due to variations in the rate of adaptive evolution, but the strength of selection acting on them. For example, we observe that ω_a_ is positively correlated to RR; we have interpreted this as evidence that the rate of adaptive evolution increases with increasing levels of recombination, but an alternative explanation is that the rate is the same, or that it decreases with RR, with the rate being more substantially overestimated in high RR genes because there are more slightly advantageous mutations; this hypothesis requires that the advantageous mutation rate is higher in high RR genes, that the mean strength of selection on advantageous mutations is lower, and that the combination of these two factors is such that the rate of adaptive substitution is lower in the high RR genes, but that the rate is sufficiently overestimated that the estimated rate of adaptive evolution is higher in high RR genes.

### Gene Function Analyses

Our analyses of VIP and non-VIP genes show that a high proportion of the variance in protein evolution in hominids is accounted for by whether or not a gene interacts with viruses, a result that corroborates Enard et al.’s (2016) findings. By disentangling the rates of adaptive and nonadaptive evolution, we find that VIP genes are under less constraint than non-VIPs, and that VIPs exhibit a higher rate of adaptive evolution. We also estimate the variance components using two-way analyses of variance, finding that the distinction between VIP and non-VIP contributes about 5× the variance in ω_a_, and 8× the variance in ω_na_ as the variation between GO categories, suggesting that whether a gene encodes a VIP has a major effect on its rate of adaptation and nonadaptation (supplementary table S1, Supplementary Material online). These results could explain why there appears to be little variation in the rate of adaptive evolution across biological functions categorized using Gene Ontology (Bierne and Eyre-Walker 2004), with viruses acting across a range of biological functions likely to be a key factor in these estimates.

Our study is likely to underestimate the amount of adaptive evolution attributable to viruses, for reasons outlined by Enard et al. (2016). Briefly, we used the categorization of VIPs and non-VIPs provided by Enard et al. (2016). However new VIPs are being discovered regularly, suggesting there are some VIPs that were not included in our analysis. Secondly, the categorization of VIP and non-VIP necessarily cannot account for proteins that adapt to viruses but do not physically interact with them (e.g., in proteins that are upstream or downstream of VIPs in signaling cascades).

### No Asymptote in the Correlation between ω_a_ and RR

Both Campos et al. (2014) and Castellano et al. (2016) found that there is a positive relationship between the rate of adaptive evolution and RR in *Drosophila*. Furthermore, Castellano et al. (2016) showed using a larger data set that the positive correlation between RR and ω_a_ asymptotes in *Drosophila*, suggesting that above a certain level of recombination Hill–Robertson interference has little effect. In this study, we do not find clear evidence for this asymptote in hominids for either the rate of adaptive or nonadaptive evolution (fig. 1*a* and supplementary fig. S1, Supplementary Material online). The lack of an apparent asymptote might be because we have few genes with high rates of recombination and so it is difficult to detect the asymptote. It might also be because the RR estimates we are using do not reflect the RR over the divergence of humans and chimpanzees. Rates of recombination evolve rapidly in hominids; humans and neanderthals share few recombination hotspots (Lesecque et al. 2014) and rates of recombination in 100-kb windows are only mildly correlated between humans and chimpanzees (Stevison et al. 2016). Hence, we may not be correlating ω_a_ against a relevant measure of the RR. The correlation in RR between humans and chimpanzees is substantially higher at the 1 Mb than the 100-kb scales (Stevison et al. 2016), so the average RR in 1-Mb windows might represent a more appropriate measure. However, we find that the ω_a_ is not significantly correlated to RR at this scale (*r* = 0.17, *P* = 0.48), whereas the correlation with ω_na_ remains significantly negatively (*r* = −0.55, *P* = 0.011). The final possibility for the lack of an apparent asymptote is that most genes are affected by HRi in hominids; that the RR in hominds is not sufficient to prevent HRi. This is perhaps not unexpected. The level of HRi will depend on several factors—the effectiveness of recombination in breaking down associations, the density of selected sites, and the mutation rate to alleles that are subject to selection; if weakly selected mutations are responsible for HRi then the effective population size and the level of nearly neutral genetic diversity will also be important. Recombination is a considerably more effective force in *Drosophila*; linkage disequilibrium decays over a scale of 10 s of base pairs (Mackay et al. 2012) rather than the 10,000 s that we observe in humans (1000 Genomes Project Consortium 2015). This 1,000-fold difference in the effectiveness of recombination is likely to more than compensate for the fact that humans have approximately 20-fold greater genome size, and a higher rate of deleterious mutation (2.1 in humans [Lesecque et al. 2012] to 1.2 in *Drosophila* [Haag-Liautard et al. 2007], respectively).

### Gene Age

Cai and Petrov (2010) found that older genes exhibit a lower rate of protein evolution (as measured by the Ka/Ks ratio) than younger genes. The authors demonstrated that this was at least in part due to stronger purifying selection acting on older genes than on younger ones, by showing that levels of nonsynonymous to synonymous polymorphism were lower in older genes. Our findings corroborate these results, with the strong negative correlation between ω_na_ and gene age showing that older genes are under a lower rate of protein evolution than younger genes. However, we also find a significant negative correlation between gene age and the rate of adaptive evolution, ω_a_, whereas Cai and Petrov found no such correlation. There are two potential causes of this discrepancy. Firstly, for this analysis Cai and Petrov group genes by their age based on lineage specificity (LS), that is, how specifically a gene and orthologs of a gene are distributed on a given phylogeny (Cai et al. 2006), whereas we group our genes by phylostratigraphic category (PL), that is, where genes are ranked by PL based on their earliest ortholog (Domazet-Loso et al. 2007). Each method has its limitations. Because the LS method relies on the phylogenetic profiles of individual genes, Cai and Petrov removed genes with patchy distributions (Cai et al. 2006), resulting in 10,032 of 20,150 genes being removed from the data set for having irregular phylogenetic profiles. The PL method relies on parsimony and assumes that a gene family can be lost, but cannot re-evolve in different lineages (Domazet-Loso et al. 2007), meaning that those genes that would be removed using the LS method are maintained in the PL method. By using the PL method, our data set contained 15,439 grouped into 19 phylostratigraphic bins. Secondly, Cai and Petrov obtained divergence and polymorphism data from the compiled Applera data set (Bustamante et al. 2005; Lohmueller et al. 2008) of 39 humans (19 African Americans and 20 European Americans), whereas we have used data from the 661 African samples within the 1000 genomes data set (1000 Genomes Project Consortium 2015). Notably, the African population has undergone a more stable demographic history than Europeans, who carry proportionally more deleterious genetic variation, which Lohmueller et al. (2008) ascribe to the bottleneck encountered by the Eurasian population at the time of the migration out of Africa. This higher proportion of segregating deleterious alleles will inevitably affect estimates of the rate of adaptive evolution, but not the ratio of nonsynonymous and synonymous substitution rates (the latter of which yields a strong correlation with gene age using both the PL and LS methods in Cai and Petrov’s study).

### The Effect of Population Contraction

It has been shown previously that the MK test can generate artifactual evidence of adaptive evolution if some nonsynonymous mutations are slightly deleterious and the population in question has undergone recent expansion, because selection is more effective during the polymorphism phase than during the divergence phase (McDonald and Kreitman 1991; Eyre-Walker 2002). Although, the effective population size in humans has increased recently, the effective population size is considerably reduced from that in the human–chimpanzee ancestor (Hobolth et al. 2007; Burgess and Yang 2008; Prado-Martinez et al. 2013; Schrago 2014). This population contraction can depress the signal of adaptive evolution in humans. Furthermore, we have shown elsewhere that if a factor, for example gene age, is correlated to the mean strength of selection against deleterious mutations, population size change will generate an artifactual correlation between that factor and the rate of adaptive evolution (Soni et al. 2021). The direction of this correlation depends on the direction of the correlation between the mean strength of selection acting against deleterious mutations and the factor in question and whether the population has expanded or contracted; for example, if factor X is positively correlated to the absolute mean strength of selection (i.e., selection is stronger against genes with larger values of X), then population contraction will induce an artifactual positive correlation between ω_a_ and X.

**Fig. 3.**
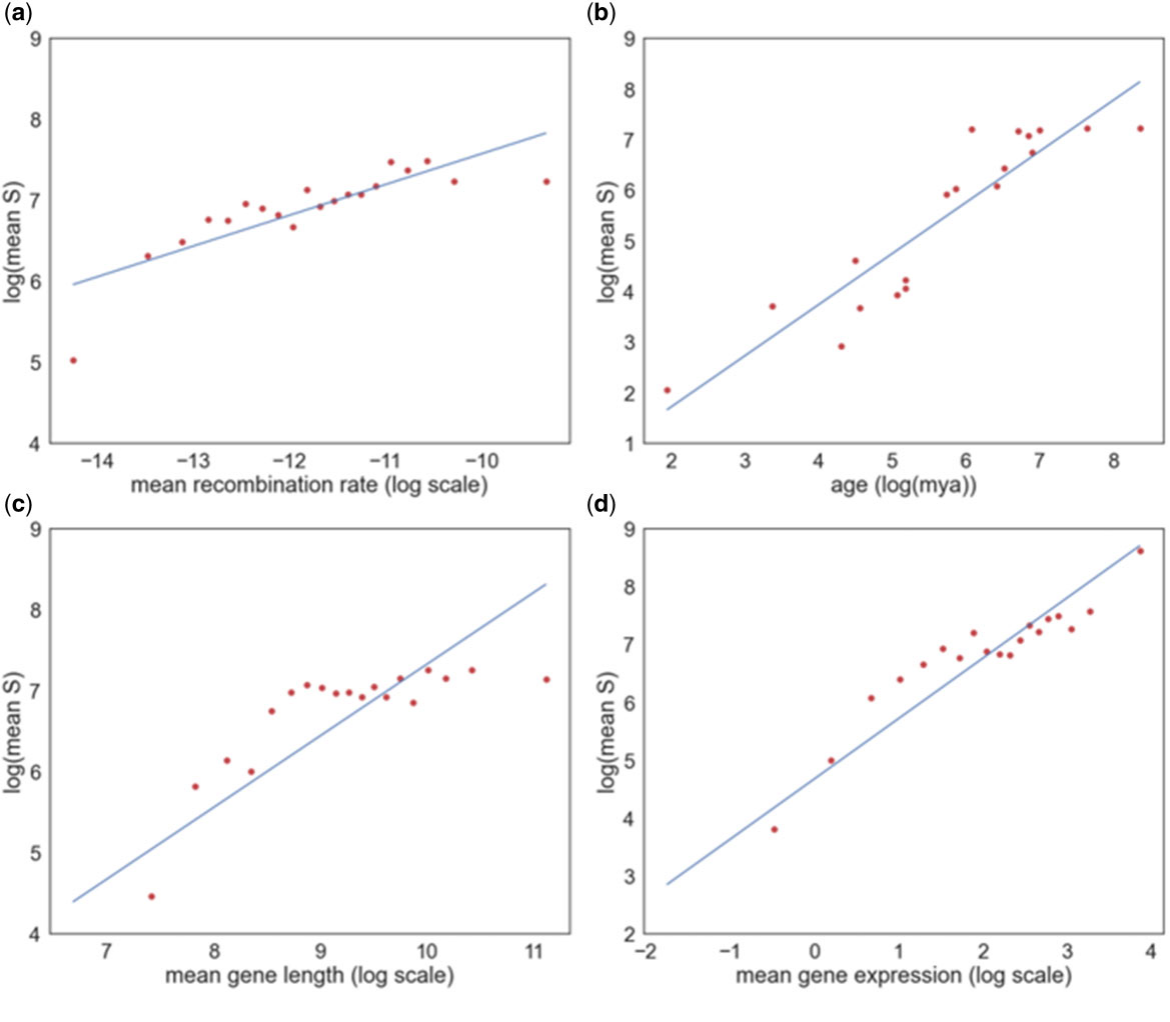
Correlation between the log of the mean strength of selection against deleterious mutations and (*a*) gene age, (*b*) RR, (*c*) gene length, and (*d*) gene expression. A linear regression has been fitted to each data set.

All four factors are positively correlated to the log absolute mean strength of selection against deleterious mutations, estimated from the site frequency spectrum (gene age: *r* = 0.916, *P* < 0.001; RR: *r* = 0.828, *P* < 0.001; gene length: *r* = 0.818, *P* < 0.001; gene expression: *r* = 0.948, *P* < 0.001) (fig. 3). Population contraction undergone by hominids should therefore tend to induce an artifactual positive correlation between ω_a_ and each factor in our analysis. This artifactual positive correlation is contrary to the negative correlation that we observe between ω_a_ and age (fig. 1). This may be one reason why we observe a weaker correlation between gene age and the rate of adaptive evolution in hominids compared with *Drosophila* and *Arabidopsis* species (Moutinho AF, Eyre-Walker A and Dutheil J, unpublished data). However, population contraction might be responsible for the positive correlation between ω_a_, RR, protein length, and expression. Because ω_na_ is estimated exclusively from polymorphism phase data, we do not expect the correlations between ω_na_ and our four factors to be affected by the population contraction.

In summary, we observe a significant correlation between the rate of adaptive evolution, RR, protein length, and gene expression, and a negative correlation between the rate of adaptive evolution and gene age. However, we cannot be very confident that any of these correlations are genuine; the positive correlation between ω_a_, RR, protein length, and gene expression might be due to an artifact of population size contraction, and the correlation between ω_a_ and age might be due to the problems of identifying rapidly evolving genes, with high values of ω_a_, in more distant taxa. In contrast, the rate of nonadaptive evolution is independently negatively correlated to all factors. We have confirmed that whether a protein interacts with viruses is an important factor in determining whether a gene undergoes high rates of adaptive and nonadaptive evolution, however, we also demonstrate that there is significant variation between GO categories, even when this factor is controlled for.

## Materials and Methods

### Data

We obtained orthologous human and chimpanzee gene sequences from the Ensembl biomart (Yates et al. 2019) for the human GRCh38 and Pan_tro_3.0 genome builds. We aligned these orthologs using MUSCLE (Edgar 2004). After filtering out genes with gaps that were not a multiple of 3, we were left with 16,344 pairwise alignments. Proportions of synonymous and nonsynonymous substitutions were estimated using codeml from the PAML package (Yang 2007) program. We used polymorphism data from the African superpopulation of the 1000 genomes data set (1000 Genomes Project Consortium 2015) to construct our site frequency spectra, with rates of adaptive (ω_a_) and nonadaptive (ω_na_) evolution estimated using Grapes (Galtier 2016), under the “GammaZero” model. We used African SNPs because the African population has been subject to relatively simple demographic processes (Gravel et al. 2011). CIs on our estimates of ω_a_ and ω_na_ were generated by bootstrapping the data set by gene.

Gene ages were obtained from Litman and Stein (2019). In this data set, genes are ranked by phylostratigraphic category (PL) based on their earliest ortholog. Gene lengths were obtained by taking the total coding sequence length of the longest transcript of each protein, whereas gene expression data were obtained from the Expression Atlas database (Papatheodorou et al. 2019), wherein the baseline experiment E-MTAB-5214 was used. These data are from the GTEx genotype-tissue expression analysis of 53 tissue samples (GTEx Consortium 2015). We estimated the arithmetic mean expression value across tissues for each gene, and binned gene by mean gene expression of 20 roughly equally sized bins (each containing 808–811 genes). RR maps were obtained from Spence and Song (2019) and Kong et al. (2010); these maps are based on population genetic and pedigree data, respectively. The mean RR was calculated between the start and end of the largest transcript for each gene, or the average RR across the MB in which the gene was centered. GO category information was obtained from Ensembl’s Biomart (Ashburner et al. 2000; Yates et al. 2019; Gene Ontology Consortium 2021).

### Correlating Factors with Rates of Adaptive and Nonadaptive Evolution

To correlate the rates of adaptive and nonadaptive evolution with each of RR, protein length, and gene expression, we binned our genes into 20 roughly equal sized bins. For gene age, we binned data by PL, of which there were 19. To control for BGC in our RR analysis, we restricted the analysis to those polymorphisms and substitutions that are unaffected by BGC—that is, A<>T and G<>C changes. This reduced our data set to about 20% of its previous size.

To investigate whether factors were independently correlated to ω_a_ and ω_na_, we ran the analysis controlling for each of the other three factors in turn. We controlled for each factor by taking the values of the co-correlate close to the modal value. We took the modal value and 0.5 standard deviations (SDs) either side which reduces the SD of the co-correlate within each analysis. Because this reduces the data set considerably, we also ran an analysis in which we predicted the correlation coefficient between Y and X under the assumption that they are only correlated to each other because they are both correlated to Z. If *r*_YZ_ is the correlation between Y and Z, then *r*_YZ_^2^ is the proportion of variance in Y explained by Z, and vice versa. Hence, the proportion of variance explained in Y by X, because of their mutual correlation to Z is *r*_YZ_^2^*r*_XZ_^2^. Hence the expected correlation coefficient between Y and X is $r_{\mathrm{YX}}=\mathrm{Sign}\;\sqrt{r_{\mathrm{YZ}}^{2}\;r_{\mathrm{XZ}}^{2}}$, where Sign is positive if both *r*_YZ_ and *r*_XZ_ are positive or negative, and negative otherwise. To assess significance, we grouped genes according to X variable, and then within each group, we generated a bootstrap data set. We estimated ω_a_, ω_na_, the mean value of X and Z for each group and the observed and predicted correlations between ω_a_, ω_na_, mean X, and mean Z. We tabulated the number of bootstrap replicates in which predicted *r*_YX_>observed *r*_YX_. We performed 100 bootstrap replicates for each analysis.

### Gene Function Analysis

Genes were divided by GO category and rates of adaptive and nonadaptive evolution were estimated for each category (note genes can contribute to multiple categories). For the VIP analysis, we split each GO category into two groups—VIP and non-VIP genes, as per (Enard et al. 2016). To test whether there was significant variation in ω_a_ and ω_na_ across GO categories, we shuffled data between gene labels; that is, for each gene, we have its synonymous and nonsynonymous site frequency spectra and numbers of synonymous and nonsynonymous substitutions. These data were randomly assigned to gene labels, hence preserving the covariance structure of the data—that is, the fact that a gene can contribute to multiple GO categories. This shuffling was performed 100 times, each time recalculating ω_a_ and ω_na_.

We are interested in the extent to which the rate of adaptive and nonadaptive evolution is determined by whether it is a VIP gene versus other GO categorizations. We can quantify this by partitioning the variance in a two-way analysis of variance where the dimensions are VIP/non-VIP, and GO category. However, to estimate the variances, we need to balance the data so that the error variance is the same for all cells in the two-way ANOVA. We did this by downsampling the data using a hypergeometric distribution, such that each cell had 200,000 combined nonsynonymous and synonymous sites. To estimate the error variance, we split the SFS and substitution data into two halves using a hypergeometric distribution and estimated ω_a_ and ω_na_ for each set; hence we have for each combination of VIP/non-VIP and GO category two estimates of the rate of adaptive and nonadaptive evolution, where the error variances for these estimates should be approximately equal.

## Supplementary Material

Supplementary data are available at *Genome Biology and Evolution* online.

## Data Availability

The analysis used publicly available data. Scripts used to process and analyze the data are available at https://github.com/vivaksoni/gene_level_factors_affecting_rates_of_evolution_in_hominids. 

## Supplementary Material

evac028_Supplementary_Data
