# Supplementary material for: Factors That Affect the Rates of Adaptive and Nonadaptive Evolution at the Gene Level in Humans and Chimpanzees
Source: Genome Biol Evol. 2022 Feb 15;14(2):evac028. doi: 10.1093/gbe/evac028 (PMC8882387; doi:10.1093/gbe/evac028)
Supplement: evac028_Supplementary_Data [file evac028_supplementary_data.docx]

**Supplementary table, S1:** Estimated variance components from two-way analysis of variance on $\omega_{a}$ for GO categories with 200,000 sites or more.

|  |  |  | **Both fixed** | **Both random** |
| --- | --- | --- | --- | --- |
| **Term** | **Number** | **MS** | **Variance** | **Variance** |
| Residual | 2 | 5.2E-05 | 0.000052 | 0.000052 |
| VIP | 2 | 0.02919 | 0.001120 | 0.001120 |
| GO | 13 | 0.00097 | 0.000229 | 0.000229 |


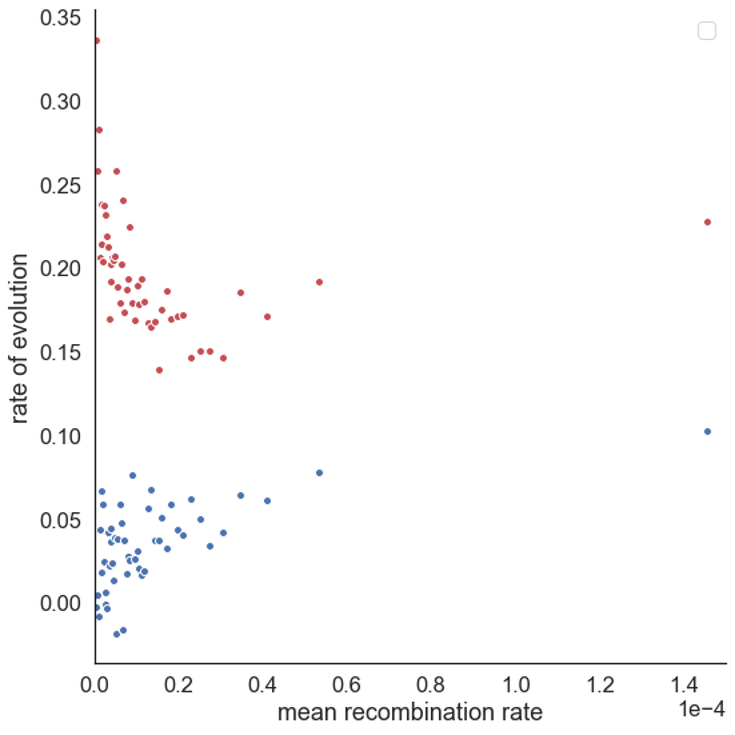


**Supplementary figure S1:** Estimates of $\omega_{a}$ and $\omega_{na}$ plotted against the log of the mean recombination rate for genes binned into 50 recombination bins of equal size. A weighted linear regression is fitted to the data, weighted by the reciprocal of the variance in the estimate of $\omega_{a}$. The variance was estimated by bootstrapping the data by gene 100 times. The respective significance of each correlation is shown in the plot legend, (*P < 0.05; **P < 0.01; ***P < 0.001; “.” 0.05 ≤ P < 0.10) for $\omega_{a}$ and $\omega_{na}$).


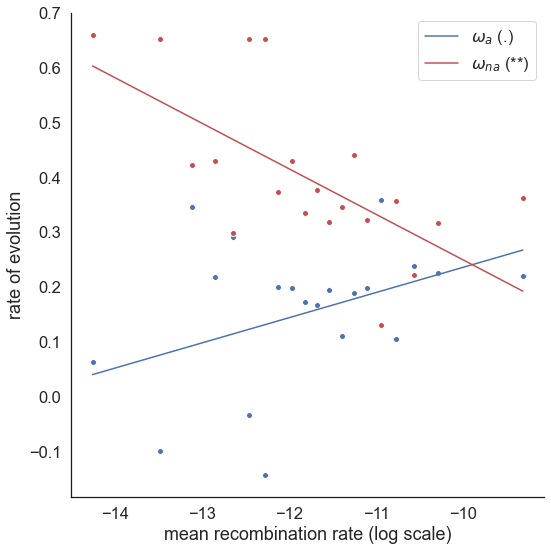


**Supplementary figure S2:** Estimates of $\omega_{a}$ and $\omega_{na}$ plotted against the log of the mean recombination rate, controlling for biased gene conversion, for genes binned into 20 recombination bins of equal size. A weighted linear regression is fitted to the data, weighted by the reciprocal of the variance in the estimate of $\omega_{a}$. The variance was estimated by bootstrapping the data by gene 100 times. The respective significance of each correlation is shown in the plot legend, (*P < 0.05; **P < 0.01; ***P < 0.001; “.” 0.05 ≤ P < 0.10) for $\omega_{a}$ and $\omega_{na}$).


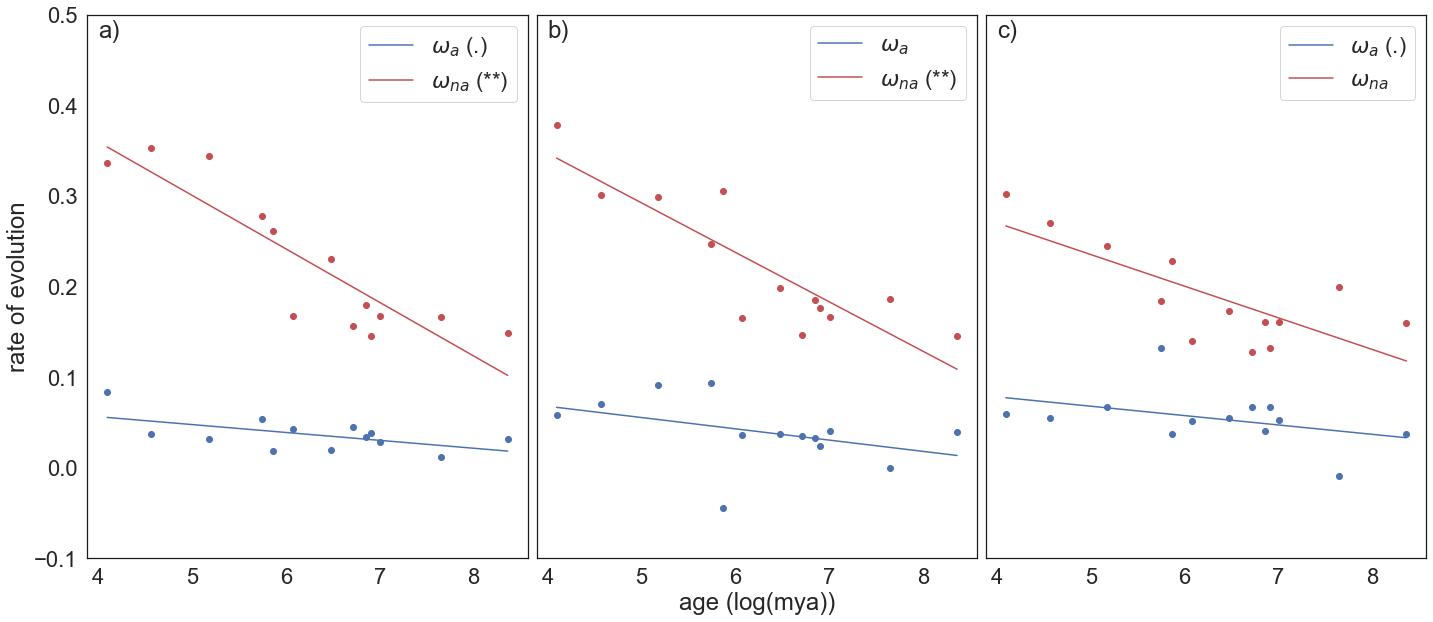


**Supplementary figure S3:** Estimates of $\omega_{a}$ and $\omega_{na}$ plotted against log gene age for genes binned into phylostratigraphic age categories, controlling for recombination rate. A weighted linear regression is fitted to the data, weighted by the reciprocal of the variance in the estimate of $\omega_{a}$. The variance was estimated by bootstrapping the data by gene 100 times. The respective significance of each correlation is shown in the plot legend, (*P < 0.05; **P < 0.01; ***P < 0.001; “.” 0.05 ≤ P < 0.10) for $\omega_{a}$ and $\omega_{na}$).


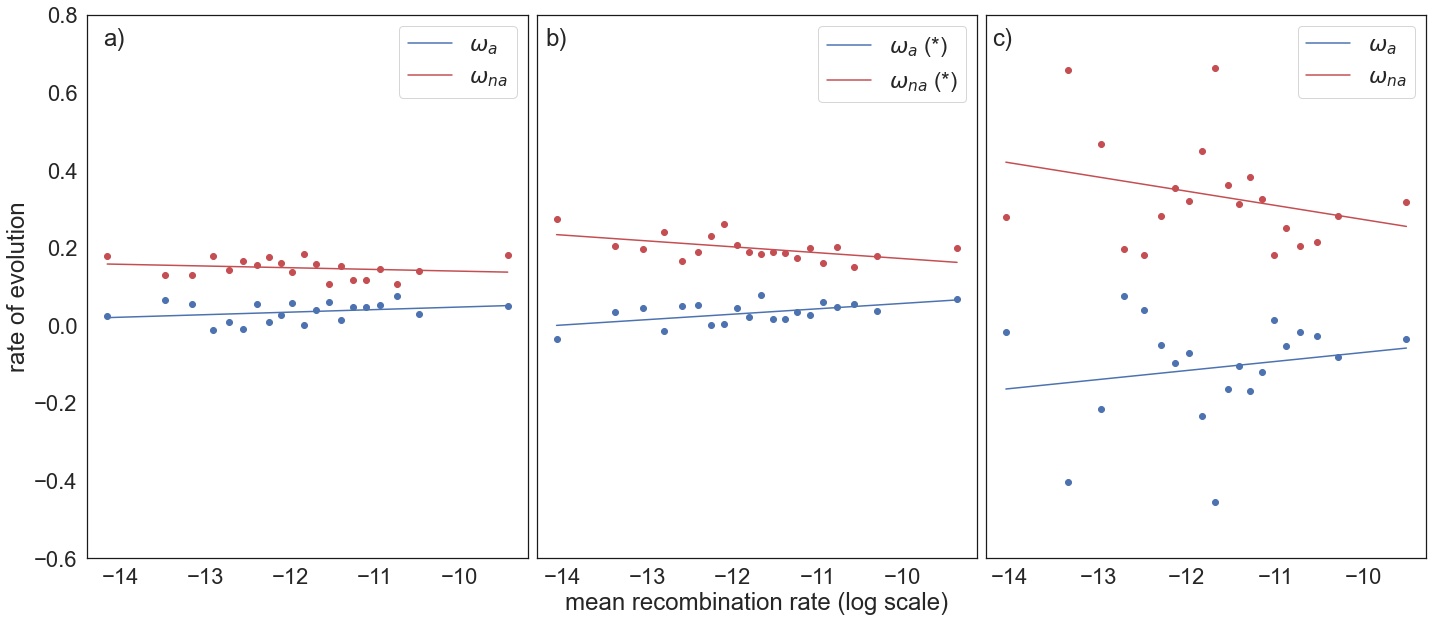


**Supplementary figure S4:** Estimates of $\omega_{a}$ and $\omega_{na}$ plotted against the log of the mean recombination rate for genes binned into 20 recombination bins of equal size, controlling for gene age. A weighted linear regression is fitted to the data, weighted by the reciprocal of the variance in the estimate of $\omega_{a}$. The variance was estimated by bootstrapping the data by gene 100 times. The respective significance of each correlation is shown in the plot legend, (*P < 0.05; **P < 0.01; ***P < 0.001; “.” 0.05 ≤ P < 0.10) for $\omega_{a}$ and $\omega_{na}$).


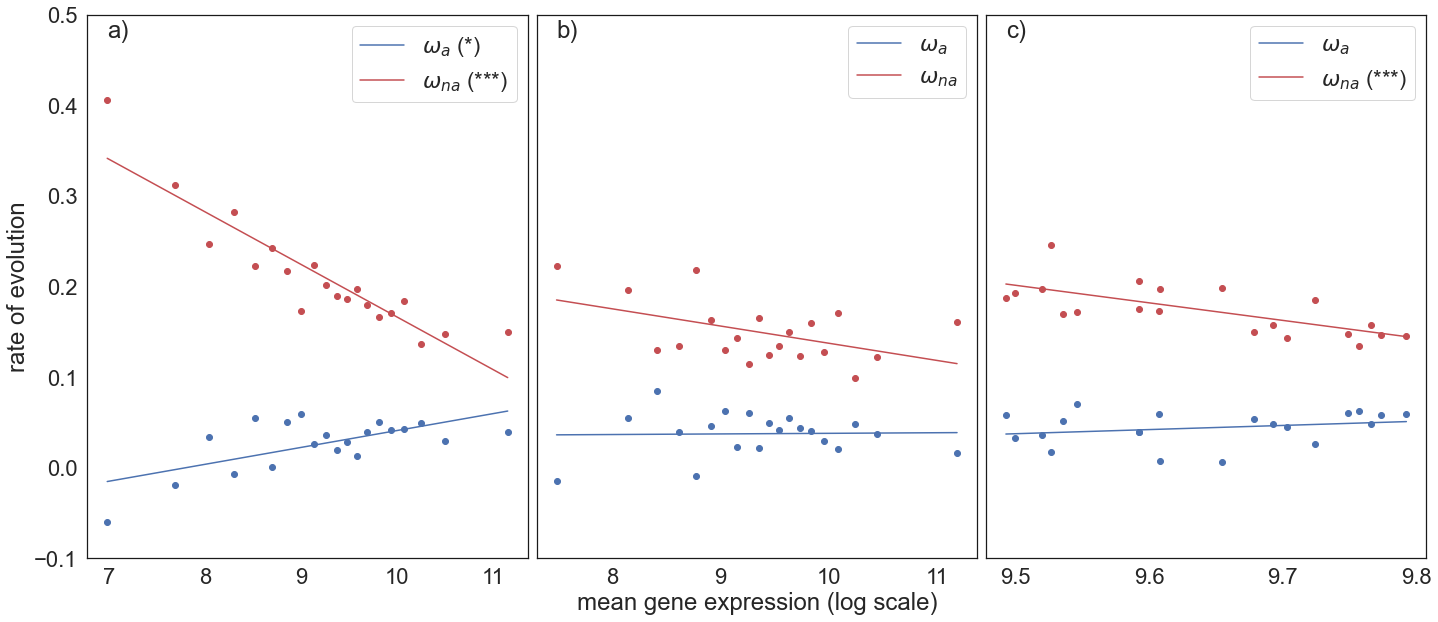


**Supplementary figure S5:** Estimates of $\omega_{a}$ and $\omega_{na}$ plotted against the log of the mean gene expression for genes binned into 20 mean expression bins of equal size, controlling for protein length. A weighted linear regression is fitted to the data, weighted by the reciprocal of the variance in the estimate of $\omega_{a}$. The variance was estimated by bootstrapping the data by gene 100 times. The respective significance of each correlation is shown in the plot legend, (*P < 0.05; **P < 0.01; ***P < 0.001; “.” 0.05 ≤ P < 0.10) for $\omega_{a}$ and $\omega_{na}$).


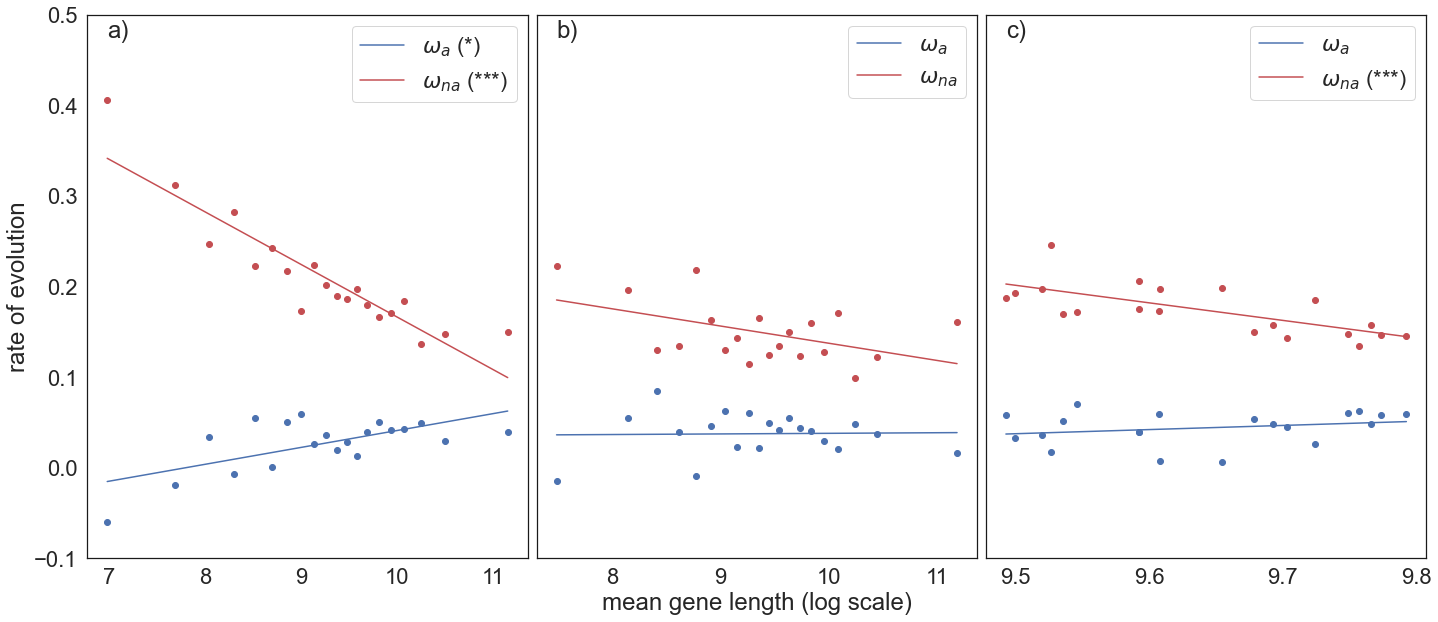


**Supplementary figure S6:** Estimates of $\omega_{a}$ and $\omega_{na}$ plotted against the log of the mean gene length for genes binned into 20 mean length bins of equal size, controlling for gene expression. A weighted linear regression is fitted to the data, weighted by the reciprocal of the variance in the estimate of $\omega_{a}$. The variance was estimated by bootstrapping the data by gene 100 times. The respective significance of each correlation is shown in the plot legend, (*P < 0.05; **P < 0.01; ***P < 0.001; “.” 0.05 ≤ P < 0.10) for $\omega_{a}$ and $\omega_{na}$).
